# Conjugated Polymer-Based Hydrogel Film for a Fast and Sensitive Detection of Fe(Ⅲ) in Vegetables

**DOI:** 10.3390/molecules29050925

**Published:** 2024-02-20

**Authors:** Xingli Ding, Li Sheng, Ge Zhang, Min Ji, Yu Li

**Affiliations:** 1 School of Environmental Science & Engineering, Tianjin University, Tianjin 300350, China; dingxingli@126.com; 2 Jiangxi Provincial Engineering Research Center for Waterborne Coatings, School of Chemistry and Chemical Engineering, Jiangxi Science & Technology Normal University, Nanchang 330013, China; shengli19980817@163.com (L.S.); zhangge20082006@126.com (G.Z.)

**Keywords:** fluorescent film, poly(9-fluorenecarboxylic acid), Fe^3+^, sodium alginate, vegetables

## Abstract

Fluorescent film sensors are ideal for the real-time outdoor detection of heavy metal ions of Fe^3+^, but they are limited because of their low sensitivity and long response time due to their special structure. In this work, we constructed a fluorescent hydrogel for the specific detection of Fe^3+^, utilizing poly(9-fluorenecarboxylic acid) (PFCA) as the sensing moiety and sodium alginate (SA) as the cross-linking substrate, which exhibited a rapid and selective recognition of Fe^3+^ among a panel of 16 anions and 21 cations. It can sense Fe^3+^ at 0.1 nM immediately owing to the porous network structure of the **PFCA-SA** film that provided enhanced ion transport channels and active sites, and the “molecular line effect” of polymer PFCA. Moreover, we successfully applied this platform to detect Fe^3+^ in four different vegetable samples. This work provides an innovative and effective strategy for fabricating green and sustainable fluorescent sensors.

## 1. Introduction

As a common but important metal, iron can be said to be the first among various metal materials, from production to application scale, called the “King of Metal” [1,2]. Importantly, iron ions participate in the transport of oxygen and nutrients, as well as the synthesis of hemoglobin and various enzymes, which are essential trace species in human and animal bodies [3,4]. Industrial production will discharge wastewater containing Fe^3+^, which will pollute resources such as water, soil, and food to varying degrees. However, an overexposure to iron can lead to serious diseases, such as hepatitis, Alzheimer’s disease, and organ dysfunction [5]. Excess iron ions especially severely threaten human health [6]. As a response to this concern, the World Health Organization (WHO) stipulates that the concentration of iron ions in drinking water should not exceed 0.3 mg/L. Thus, the detection of iron ions is very important, and the exploration of accurate and rapid detection methods are being carried out.

Traditional detection methods for the detection of iron ions mainly include inductively coupled plasma mass spectroscopy (ICP-MS), inductively coupled plasma atomic emission spectrometry (ICP-AES), and atomic absorption, which can accurately analyze the concentration of iron ions in real samples. But these methods rely heavily on expensive equipment, complicated operation, and strong laboratory dependence [7,8,9]. The fluorescence analytical method stands out because it overcomes the problems of traditional detection methods. Fluorescent sensors especially display high sensitivity and their selectivity can be modulated by rational molecular design. Among them, fluorescent film sensors have adjustable shape and size, easy driveability, good stability and portability, and non-invasive, which are very suitable for outdoor detection [10,11,12]. In 2022, they have been recognized as one of the top ten emerging chemical technologies by the International Union of Pure and Applied Chemistry [13]. Researchers have devoted most efforts to the development of new materials for various targets. For detecting iron ions, some good film sensors have been reported, as listed in Table 1 [14,15,16,17,18,19,20,21,22,23,24]. Regardless of the sensors being solution sensors or film sensors, selectivity, sensitivity, and response time are three important parameters. From Table 1, we can see that these film sensors with or without substrates can selectively target iron ions among other metal ions and some of them also exclude the interferences of common anions. From this aspect, these sensors meet the selectivity requirement. After analyzing their sensitivity, we concluded that most of these sensors do not achieve high sensitivity, while their limit of detections (LODs) could only reach μM. Only one film realizes the detection at the 10^−8^ M level [22]. More importantly, there are three films that can detect iron ions within several minutes [14,17,21] and we could not evaluate the practicability in real samples using other sensors. In other words, although film sensors display some remarkable advantages in comparison to solution sensors, they are limited in detection sensitivity and response time, which are two very important parameters for sensors. Hence, their applications are only focused on water samples [15,16,18,19,23]. Compared with solution sensors, film sensors have a lower sensitivity and longer response time because film sensing involves complex surface interface interactions. This generally consist of energy transfer and mass transfer. (1) The “energy transfer” process refers to the interaction between the sensing unit and the analyte molecules, which affects the energy change in the luminous process of the sensing unit. (2) The process of mass transfer refers to the adsorption, diffusion, and desorption of analyte molecules in the fluorescence active layer. It can be seen that the sensing performance of thin films mainly depends on the sensing unit that determines the sensing mechanism and the active layer structure that affects the adsorption, resolution, and diffusion of analyte molecules. As seen in Table 1, these films mainly contain quantum dots and organic molecules. These materials as sensing units need to be improved. In addition, unlike for a solution, ions have great resistance to penetrate films, which will undoubtedly delay the response time and lower the sensitivity. Thus, the development of sensitive film sensors for fast-detecting iron ions is essential but presents great challenges.

**Scheme 1:**
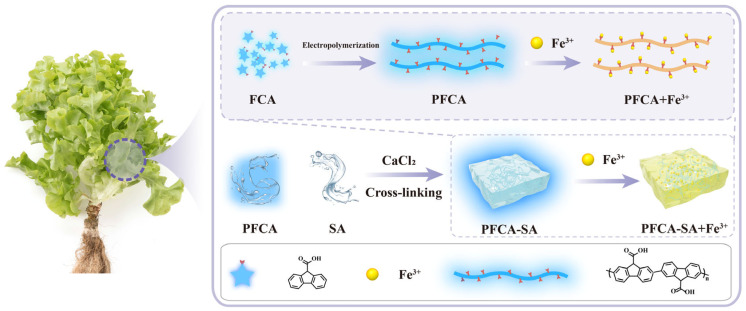
Preparation of the **PFCA-SA** hydrogel film and determination of Fe^3+^ in real samples.

This work presents the development of a highly sensitive film sensor that employs a fluorescent conjugated polymer as its foundation. The main objective of this sensor is to identify the presence of Fe^3+^ in vegetables. To enhance the sensitivity of the film sensor, we utilized electrosynthesized poly(9-fluorenecarboxylic acid) (PFCA) as the sensing unit, capitalizing on its exceptional “molecular line effect”. A hydrogel with a three-dimensional (3D) porous network structure prepared from food grade sodium alginate (SA) cross-linking was as the film substrate for accelerate the contact of ions with the sensing unit. The resulting fluorescent film, **PFCA-SA**, demonstrated remarkable selectivity toward Fe^3+^ in comparison to the 21 other metal ions and 16 anions. Importantly, it exhibited the ability to promptly detect Fe^3+^ in actual vegetable samples, with an LOD as low as 0.10 nM (Scheme 1).

## 2. Results and Discussion

### 2.1. Electrosynthesis and Characterization of PFCA

**Figure 1:**
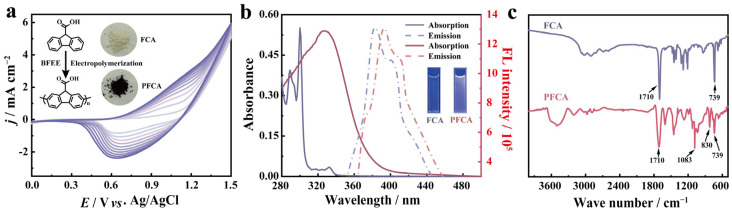
(**a**) The CVs of FCA. The potential scan rate of 100 mV s^−1^. (**b**) Spectrograms of FCA and PFCA in DMSO. (**c**) FT-IR spectra of FCA and PFCA.

Significant advancements have been made in the synthesis and application of π-conjugated materials since the discovery of conductive polymers (CPs). These CPs have garnered widespread attention due to their low-cost nature and potential in optoelectronic devices. They possess strong light capturing abilities and can enhance fluorescence sensing signals. Additionally, they exhibit high optical and electronic properties, are easily processed in solution, and offer flexibility in composition. When these CPs are employed as fluorescent sensors, they exhibit a very high sensitivity for targets due to their “molecular line effect”. In other words, CPs are good candidates for constructing sensors with high sensitivity. Polypyrene (PF) is a highly esteemed example of a conjugated polymer (CP) owing to its exceptional light absorption and optoelectronic performance. As a result, PF exhibits immense potential in various fields such as display technology, solar cells, lasers, and transistors. Nevertheless, it is imperative to address the issue of aggregation quenching (ACQ) that arises from the π–π stacking interaction between PF molecules. To mitigate this effect, a commonly employed approach involves the incorporation of side chain moieties on the C-9 position of fluorene. Our previous scientific investigation has successfully demonstrated the utilization of carboxyl functional groups on the C-9 position of fluorene, resulting in the unique ability of PFCA (perfluorinated carboxylic acid) to selectively detect and identify Fe^3+^ through a one-step electrochemical synthesis. This significant achievement highlights the potential of modifying the C-9 position of fluorene with carboxyl groups to enhance the sensing capabilities of PFCA toward Fe^3+^ [25]. The resulting polymer exhibits minimal impact from the solvent effect of ionic solvent, making it a promising hydrogel film sensing unit. In this work, we chose 9-fluorenecarboxylic acid (FCA) as the polymerization precursor to prepared polymer film because we have found that the polymer PFCA displayed very high selectivity and sensitivity to Fe^3+^ in solution. The electrochemical experiments of monomer FCA were carried out in boron trifluoride diethyl etherate (BFEE) solution with a concentration of 0.02 M. The cyclic voltammograms (CVs) of FCA showed increased redox current densities with the increasing cycle number of potential scans, implying that the polymer film was gradually formed (Figure 1a). In addition, visual inspection also indicated that a dark brown polymer film was formed on the surface of the working electrode. As shown in Figure 1a, the color of monomer FCA was faint yellow and changed to dark brown when it was polymerized.

To further demonstrate the successful preparation of PFCA, we studied the absorption and emission spectra of monomer and polymer. As shown in Figure 1b, the monomer FCA showed two absorption peaks at 289 nm and 300 nm, which were attributed to the characteristic absorption of fluorene. In comparison, the absorption spectrum of polymer PFCA exhibited one broad band with a peak at 328 nm. In addition, the emission spectrum of PFCA also displayed a red shift, which is mainly attributed to the increased length of the conjugated chain. This shift showed that an increase in the conjugation length of the polymer PFCA relative to the monomer FCA, which further showed that the monomer had been polymerized into polymer.

We also studied the FT-IR spectra of the monomer and polymer to confirm the polymerization sites of PFCA (Figure 1c). For the monomer, the peak at around 1710 cm^−1^ was attributed to the stretching vibration of a C=O group, while this peak was also retained in PFCA. The peak at 739 cm^−1^ was a characteristic peak of in-plane and out-of-plane C-H bending modes of 1,2-disubstituted benzene in FCA and PFCA. But in the spectrum of PFCA, a peak appeared at about 830 nm, which was attributed to a 1,2,4-trisubstituted benzene ring. This indicates that the polymerization sites of FCA were located at 2 and 7 positions. Subsequently, the polymer film was prepared using electrodeposition for subsequent detection and application.

### 2.2. Preparation and Characterization of PFCA-SA Hydrogel Films

The obtained polymer PFCA was power, so we needed to employ a substrate to prepare the film sensor. In line with the sustainable development strategy, the environmentally friendly SA was selected as the hydrogel substrate, which is a well-established polymer used in film preparation. SA can not only effectively combine with various substances, including carbon materials and organic compounds, but can also be degraded after being cross-linked into a hydrogel. The utilization of degradable fluorescent film sensors proves instrumental not only for facilitating the real-time detection of heavy metal ions in outdoor environments, but also mitigating the risk of secondary contamination post-use. Thus, the prepared **PFCA-SA** hydrogel films will align seamlessly with the imperatives of a sustainable development strategy, emphasizing the pressing need for innovative and environmentally conscious solutions in the field of pollutant detection and management. In addition, when SA is cross-linked into a hydrogel, it will exhibit a porous structure. This hydrogel configuration imparts an increased number of ion transport channels and active sites, facilitating the exchange of materials within the surrounding water setting.

**Figure 2:**
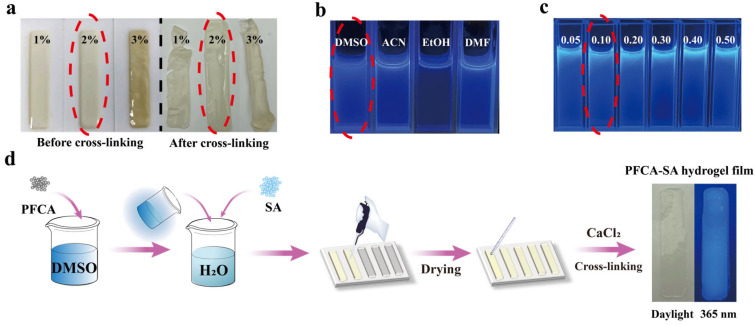
(**a**) Hydrogel films of SA with different concentrations. (**b**) The fluorescence pictures of PFCA in different solvents (DMSO, ACN: acetonitrile, EtOH: ethanol, DMF: N,N-dimethylformamide). (**c**) The fluorescence pictures of PFCA with different concentrations in DMSO. (**d**) The preparation process of the **PFCA-SA** hydrogel film. (The red circles indicate the selected SA concentration, solvent and PFCA concentration).

The initial objective of this study was to assess the concentration of SA in order to ensure the high quality of hydrogel films. During the process of forming the films, SA with different concentrations was prepared in water, and it was noted that using a 2 wt% SA concentration yielded favorable processability (Figure 2a). Additionally, the hydrogel film formed through crosslinking with this concentration of SA exhibited remarkable flexibility and strength, without being susceptible to breakage. In terms of the PFCA prepared through electrodeposition, it demonstrated excellent solubility in four commonly used organic solvents, while also emitting a striking deep blue fluorescence in dimethyl sulfoxide (DMSO) (Figure 2b). To mitigate the ACQ effect, the optimal concentration of PFCA was investigated under a 365 nm ultraviolet lamp. It was found that the fluorescence was the strongest when the PFCA content was 0.1 g/L in DMSO (Figure 2c). To prepare the hydrogel, PFCA dissolved in DMSO and SA were added into water, and then the mixture was transferred into a mold. After using a 0.1 M CaCl_2_ solution (Figure 2d), a transparent and flexible hydrogel film was obtained (Supplementary Video S1) which could emit stable blue fluorescence at 365 nm.

The internal structures of the SA hydrogel and **PFCA-SA** hydrogel film were studied using a scanning electron microscope (SEM). As shown in Figure S1a, the SA hydrogel substrate exhibited porous morphology, which was the skeleton formed by the coordination bond between -COO^−^ and Ca^2+^ in the sodium alginate, along with the 1,4-glycosidic bond between the two aldehydes contained in sodium alginate. Upon the addition of PFCA, the **PFCA-SA** film displayed a more pronounced box structure (Figure S1b), which effectively exposed a huge interface for the interaction between PFCA and ions.

### 2.3. Detection of Fe^3+^ Using PFCA-SA Hydrogel Films

**Figure 3:**
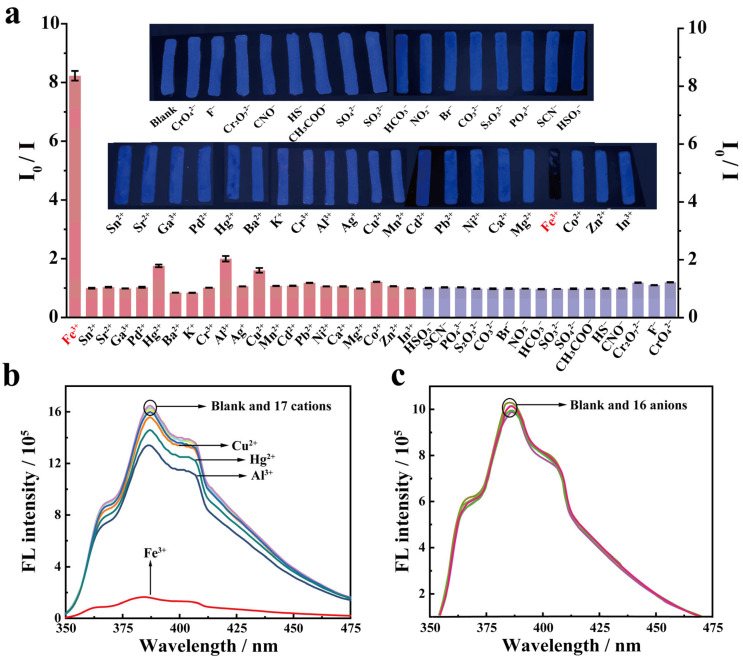
(**a**) Fluorescence response of the **PFCA-SA** hydrogel film to different anions (insert: photo of selectivity of the **PFCA-SA** hydrogel film to different anions and cations under 365 nm UV Lamp). (**b**,**c**) Fluorescent spectra of the **PFCA-SA** hydrogel film to different cations and anions.

It is known that achieving a high selectivity for the analyte of interest over a complex background of potentially competing species is a challenging task in sensor development. To ascertain whether the **PFCA-SA** hydrogel film shares this unique recognition ability for Fe^3+^, we studied its selectivity. As depicted in Figure 3, the introduction of the Fe^3+^ ion immediately quenched the fluorescence intensity of the **PFCA-SA** hydrogel film. In contrast, the presence of other metal ions and anions induced only a small portion of fluorescence quenching. This showed that the PFCA-SA hydrogel film had a high selectivity to Fe^3+^. Remarkably, the quenching was instantaneous (Supplementary Video S2), akin to the response time observed in liquid form. Compared with other film sensors reported (Table 1), the PFCA-SA hydrogel film displayed a very short time, which benefited from the porous structure of the SA hydrogel.

**Figure 4:**
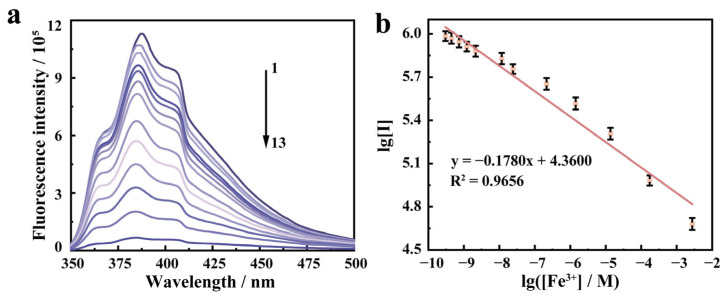
(**a**) Fluorescence emission spectra of the **PFCA-SA** hydrogel film toward Fe^3+^ with different concentrations ((1) blank; (2) 3.03 × 10^−10^ M; (3) 4.47 × 10^−10^ M; (4) 7.34 × 10^−10^ M; (5) 1.21 × 10^−9^ M; (6) 2.15 × 10^−9^ M; (7) 1.17 × 10^−8^ M; (8) 2.42 × 10^−8^ M; (9) 2.15 × 10^−7^ M; (10) 1.42 × 10^−6^ M; (11) 1.36 × 10^−5^ M; (12) 1.74 × 10^−4^ M; (13) 6.01 × 10^−3^ M). (**b**) linear plots of their fluorescence intensity against the Fe^3+^ concentration (Ex = 330 nm).

The recognition ability of the **PFCA-SA** hydrogel film toward Fe^3+^ was studied experimentally using the fluorescence method. When the concentration of Fe^3+^ was in the range of 0.303 nM–6.01 mM (Figure 4), it showed a perfect linear relationship (y = −0.1780x + 4.3600 (R^2^ = 0.9656)). According to the formula whereby LOD = 3 s/m, the LOD for Fe^3+^ achieved by the **PFCA-SA** hydrogel film was determined to be 0.1 nM, representing a very low LOD (Table 1). This rapid response time and high sensitivity are the result of the joint action of the SA substrate with a porous structure and the sensing unit PFCA with a “molecular line effect”. These results indicated that the **PFCA-SA** hydrogel film has the capability to detect Fe^3+^ in real samples.

### 2.4. Application of the PFCA-SA Hydrogel Film

According to the dietary pyramid, people should consume a variety of colors and types of vegetables every day to ensure access to various nutrients. The root system of vegetables is the main organ that absorbs water and nutrients from the soil, and its development has a direct impact on the growth, development, and yield of vegetables. When plants absorb water containing excessive Fe^3+^, it not only affects their own growth, but also causes irreversible damage to human health by consuming vegetables containing excessive Fe^3+^. Therefore, the rapid and sensitive detection of Fe^3+^ concentration in vegetable samples holds paramount importance. In this investigation, the standard addition method was used to assess the presence of Fe^3+^ in four vegetables, hence evaluating the feasibility of our proposed method. The results were expressed by linear equations, with all measurements repeated three times. As shown in Table 2, none of the real samples caused significant fluorescence quenching of the **PFCA-SA** film, indicating that these samples may not contain Fe^3+^. Then, solutions containing Fe^3+^ at varying concentrations were added to the four vegetable samples and the resulting fluorescence intensities of the **PFCA-SA** film were recorded. Their recoveries were calculated based on linear laws. These satisfactory detection outcomes, high recovery rates (98.94–100.82%), and RSD (0.20–1.93%) in these samples affirmed that **PFCA-SA** film could detect Fe^3+^ in vegetables. Recoveries and RSD values of the sensor within an acceptable range indicated that the **PFCA-SA** film could be feasible for practical application without the sample purification step and other complex processes, even though in complex matrices.

## 3. Experimental Section

### 3.1. Chemicals and Materials

FCA (97%, Aladdin, Shanghai, China) and SA (Biochemical grade, Aladdin) were used directly. BFEE (Beijing Changyang Chemical Plant, Beijing, China) was purified by distillation before use. CaCl_2_ (AR) was purchased from Xilong Chemical Co., Ltd., Shantou, China. Both DMSO (AR, 99.8%) and ethyl acetate (EA, AR, 99.5%) were purchased from Shanghai Titan Scientific Co., Ltd., Shanghai, China. All other chemicals and reagents (analytical grade) were used directly without further purification.

The aqueous solution of Sn^2+^ was prepared from its chloride salt. The aqueous solution of Ag^+^ was prepared from its perchloric acid salt. Aqueous solutions of Sr^2+^, Ga^3+^, Pd^2+^, Hg^2+^, Ba^2+^, K^+^, Cr^3+^, Al^3+^, Cu^2+^, Mn^2+^, Cd^2+^, Pb^2+^, Ni^2+^, Ca^2+^, Mg^2+^, Fe^3+^, Co^2+^, Zn^2+^, and In^3+^ were prepared from their nitrate salts. The aqueous solution of CrO_4_^2−^ and Cr_2_O_7_^2−^ was prepared from their kalium salts. T aqueous solutions of F^−^, CNO^−^, HS^−^, CH_3_COO^−^, SO_4_^2−^, SO_3_^2−^, HCO_3_^2−^, NO_2_^−^, Br^−^, CO_3_^2−^, S_2_O_3_^2−^, PO_4_^3−^, SCN^−^, and HSO_3_^−^ were prepared from their sodium salts.

### 3.2. Instrumentation

Electrochemical polymerization was performed using a Versa Stat 3 electrochemical workstation (EG&G Princeton Applied Research) under computer control. All infrared tests were completed using the PerkinElmer FT-IR spectrometer. Absorption spectra were recorded using an Agilent 8454 UV-vis spectrophotometer. Fluorescence experiments were carried out using an Edinburgh FS5 steady-state transient fluorescence spectrometer. The internal structure of the samples was observed via SEM (Apreo 2 produced by Thermo Fisher Scientific, Waltham, MA, USA).

### 3.3. Methods

#### 3.3.1. Preparation of PFCA

The electrochemical polymerization of FCA was performed in a one-compartment cell using a potentiostat–galvanostat under computer control. ITO glass was selected as the working electrode, platinum wire as the counter electrode, and self-made Ag/AgCl as the reference electrode. Before each experiment, the above electrodes need to be carefully polished with sandpaper (1500 mesh), cleaned with water and acetone, and then dried in air. The thin film grows steadily at a voltage of 1.40 V vs. Ag/AgCl, and its thickness is controlled by the total charge of the battery, which is directly read from the current time (I-t) curve by a computer. The obtained polymer films need to be repeatedly washed with anhydrous ether to remove electrolytes and monomers. In spectral analysis, the PFCA film is doped with a dilute HCl solution for 2 days, the doped ions are removed, and then washed repeatedly with pure water. Finally, these films were vacuum-dried at 60 °C for 24 h.

#### 3.3.2. Preparation of the PFCA-SA Hydrogel Film

PFCA was dissolved in DMSO to prepare a fluorescent solution with a concentration of 0.1 g/L. At the same time, a solution was prepared by adding 0.44 g of SA to 22 mL of deionized water at a constant temperature of 70 °C. Subsequently, the PFCA fluorescence solution was mixed with the SA solution (*v:v* = 9:11) to obtain a homogenous mixture solution. The mixed solution was sucked into the mold using a syringe and dried at 65 °C for 20 min. The dried hydrogel films were cross-linked with 0.1 M CaCl_2_ solution for 2 min to obtain the **PFCA-SA** fluorescent films. Upon the addition of a CaCl_2_ solution, the G unit within the alginate macromolecule could bind with Ca^2+^, leading to the formation of a water-insoluble hydrogel with a stable three-dimensional “egg box structure”.

#### 3.3.3. Preparation of Real Samples

The four vegetable samples tested in this work were purchased from local supermarkets and served as the subjects of our analysis. To prepare these samples for testing, the vegetable samples were centrifuged after juice extraction. The resulting supernatant was utilized for subsequent experiments. The practical applicability of the **PFCA-SA** hydrogel film in real samples was evaluated through the application of the standard addition method. This involved adding varying concentrations of Fe^3+^ to these vegetable samples. Subsequently, all sample solutions containing Fe^3+^ or the controls without Fe^3+^ were detected using the **PFCA-SA** hydrogel film.

## 4. Conclusions

In summary, a fluorescent hydrogel film using conjugated polymer PFCA as the recognition unit and cross-linked SA as the substrate has been prepared for the detection of Fe^3+^. The prepared **PFCA-SA** film could immediately sense Fe^3+^ with a LOD of 0.1 nM, and it showed excellent selectivity for Fe^3+^ among 16 anions and 21 cations. Then, it was successfully used in four vegetables. The selectivity of the **PFCA-SA** hydrogel film toward Fe^3+^ has been rigorously confirmed through extensive testing involving various metal ions and anions. It is worth emphasizing that the real-time response of the thin film to Fe^3+^ is comparable to that of liquid-phase sensors, thus showcasing its practicality. This endeavor underscores the potential to tackle the challenges posed by heavy metal ion pollution across a wide range of applications.

## Figures and Tables

**Table 1:** The parameters and performance of different film sensors for Fe^3+^ detection.

Sensor	Substrate	Response Time	Working Concentration Range	LOD	Interference Exclusion	Applications
P5CI [14]	ND	5 min	0.2 μM–0.5 mM	0.897 ppb	Metal ions, anions, natural amino acids, organic acids, and carbohydrates	ND
PYTPAG2 [15]	ND	ND	0–40 μM	0.5 μM	Metal ions	Natural water
P-PAH [16]	ND	ND	1–8.0 mM	74.5 μM	Metal ions and anions	Sea, tap, and drinking water
R6G–PPP [17]	Cellulosic paper	1 min	6.3 μM–0.1 mM	1 μM	Metal ions	ND
PAZU [18]	ND	ND	1–8 mM	86.15 μM; 28.90 μM	Metal ions	Drinking, sea, and tap water
N-GQDs [19]	ND	ND	0.5 μM–50 μM	ND	Metal ions	Tap water
WBPU-N-C-dots [20]	ND	ND	0–0.2 mM	2.19 μM	Metal ions	ND
Eu_0.24_Tb_0.76_-BHM-COOH [21]	Polylactic acid	5 min	ND	4.47 μM	Metal ions	ND
Tfpa–Mth COF [22]	Quartz crystalmicrobalance chip	ND	0–0.1 mM	64 nM	Metal ions and anion	ND
SQDs [23]	Poly(vinyl alcohol)	ND	0–0.53 mM	0.69 μM	Metal ions	Tap water and river water
H_3_TATAB [24]	Poly(methyl methacrylate)	ND	ND	68 ppb	Metal ions	ND
PFCA [In this work]	SA	Immediately	0.303 nM–6.01 mM	0.10 nM	Metal ions and anions	Vegetables

ND: not discussed.

**Table 2:** Determination of Fe^3+^ in vegetable samples using the **PFCA-SA** film.

Sample	Fe^3+^ Spiked (M)	Fe^3+^ Found ($\bar{x}$ ± SD) (M)	Recovery (%)	RSD (%)
Wild rice stem	-	-	-	-
	4.90 × 10^−6^	(4.93 ± 0.01) × 10^−6^	100.61	0.20
	4.95 × 10^−5^	(4.97 ± 0.02) × 10^−5^	100.40	0.40
	4.70 × 10^−4^	(4.71 ± 0.04) × 10^−4^	100.21	0.85
Water spinach	-	-	-	-
	4.90 × 10^−6^	(4.93 ± 0.02) × 10^−6^	100.61	0.41
	4.95 × 10^−5^	(4.97 ± 0.03) × 10^−5^	100.40	0.60
	4.70 × 10^−4^	(4.65 ± 0.03) × 10^−4^	98.94	0.65
Cabbage	-	-	-	-
	4.90 × 10^−6^	(4.94 ± 0.07) × 10^−6^	100.82	1.42
	4.95 × 10^−5^	(4.92 ± 0.02) × 10^−5^	99.39	0.41
	4.70 × 10^−4^	(4.67 ± 0.09) × 10^−4^	99.36	1.93
Celery	-	-	-	-
	4.90 × 10^−6^	(4.92 ± 0.02) × 10^−6^	100.41	0.41
	4.95 × 10^−5^	(4.90 ± 0.06) × 10^−5^	98.98	1.22
	4.70 × 10^−4^	(4.66 ± 0.01) × 10^−4^	99.15	0.21

## Data Availability

Data are contained within the article and Supplementary Materials.

## Supplementary Materials

The following supporting information can be downloaded at: https://www.mdpi.com/article/10.3390/molecules29050925/s1, Video S1: The obtained transparent and flexible **PFCA-SA** hydrogel film; Video S2: Fluorescence sensing experiment of **PFCA-SA** hydrogel film with and without Fe^3+^ solution; Figure S1. SEM of SA (a) and **PFCA-SA** hydrogel film (b).
