# Supplementary material for: Conjugated Polymer-Based Hydrogel Film for a Fast and Sensitive Detection of Fe(Ⅲ) in Vegetables
Source: Molecules. 2024 Feb 20;29(5):925. doi: 10.3390/molecules29050925 (PMC10934421; doi:10.3390/molecules29050925)
Supplement: Supplementary file 1 [file molecules-29-00925-s001.zip › molecules-2847792-Figure S1.pdf]

Supporting Information

Conjugated Polymer-Based Hydrogel Film for a Fast and
Sensitive Detection of Fe(III) in Vegetables

Xingli Ding [1,†], Li Sheng [2,†], Ge Zhang [2], Min Ji [1,*] and Yu Li [2,*]

[1] School of Environmental Science & Engineering, Tianjin University, Tianjin 300350, China; dingxingli@126.com
[2] Jiangxi Provincial Engineering Research Center for Waterborne Coatings, School of Chemistry and
Chemical Engineering, Jiangxi Science & Technology Normal University, Nanchang 330013, China;
shengli19980817@163.com (L.S.); zhangge20082006@126.com (G.Z.)
[*] Correspondence: jimin@tju.edu.cn (M.J.); liliyuly@163.com (Y.L.)
[†] These authors contributed equally to this work.

Video S1 The obtained transparent and flexible **PFCA-SA** hydrogel film.

Video S2 Fluorescence sensing experiment of **PFCA-SA** hydrogel film with and without Fe^{3+} solution.

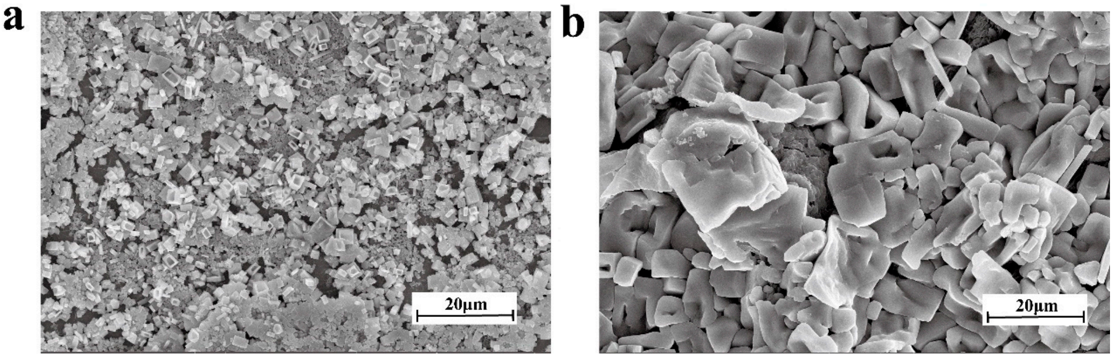

Figure S1. SEM of SA (a) and **PFCA-SA** hydrogel film (b).
